# Prognostic and predictive significance of podocalyxin-like protein expression in pancreatic and periampullary adenocarcinoma

**DOI:** 10.1186/s12907-015-0009-1

**Published:** 2015-05-30

**Authors:** Margareta Heby, Jakob Elebro, Björn Nodin, Karin Jirström, Jakob Eberhard

**Affiliations:** Department of Clinical Sciences Lund, Division of Oncology and Pathology, Lund University, Skåne University Hospital, 221 85 Lund, Sweden

**Keywords:** Periampullary adenocarcinoma, Pancreatic cancer, Podocalyxin-like 1, Immunohistochemistry, Biomarkers, Prognosis, Response prediction

## Abstract

**Background:**

Adenocarcinoma of the periampullary region is associated with poor prognosis and new prognostic and treatment predictive biomarkers are needed for improved treatment. Membranous expression of podocalyxin-like 1(PODXL), which is a cell-adhesion glycoprotein and stem cell marker, has been found to correlate with an aggressive tumour phenotype and adverse outcome in several cancer types. The aim of the present study was to examine the clinicopathological correlates, prognostic and predictive significance of tumour-specific PODXL expression in a retrospective cohort of pancreatic and periampullary carcinoma, morphologically divided into intestinal type (I-type) and pancreatobiliary type (PB-type) tumours.

**Methods:**

Immunohistochemical expression of PODXL was analysed in tissue microarrays with primary tumours and a subset of paired lymph node metastases from 175 patients operated with pancreaticoduodenectomy for periampullary adenocarcinoma. Chi square test was applied to analyse the relationship between PODXL expression and clinicopathological parameters. Kaplan Meier analysis and Cox regression models were applied to estimate differences in 5-year overall survival (OS) and recurrence-free survival (RFS) in strata according to membranous and non-membranous PODXL expression.

**Results:**

Membranous PODXL expression was significantly higher in primary PB-type (49.5 %) as compared with I-type (17.5 %) tumours. In PB-type tumours, PODXL expression was significantly associated with female sex (*p* = 0.005), location to the pancreas (*p* = 0.005), and poor differentiation grade (*p* = 0.044). Membranous PODXL expression was significantly associated with a reduced RFS (HR = 2.44, 95 % CI 1.10–5.44) and OS (HR = 2.32, 95 % CI 1.05–5.12) in I-type tumours and with a reduced RFS (HR = 1.63, 95 % CI 1.07–2.49) but not OS in PB-type tumours. PODXL remained a significant independent prognostic factor only in I-type tumours (HR = 5.12, 95 % CI 1.43–18.31 for RFS and HR = 7.31, 95 % CI 2.12–25.16 for OS). Patients with I-type tumours displaying membranous PODXL expression had a significant beneficial effect of adjuvant chemotherapy regarding 5-year OS.

**Conclusion:**

Membranous expression of PODXL is significantly higher in PB-type than in I-type periampullary adenocarcinomas and an independent factor of poor prognosis in the latter. The results further indicate a beneficial effect of adjuvant chemotherapy on I-type tumours with membranous PODXL expression, suggesting the potential utility of PODXL as a biomarker for improved treatment stratification of these patients.

**Electronic supplementary material:**

The online version of this article (doi:10.1186/s12907-015-0009-1) contains supplementary material, which is available to authorized users.

## Background

Adenocarcinoma of the periampullary region, including tumours originating in the distal bile duct, pancreas, ampulla of Vater and the periampullary duodenum, are a heterogeneous group of neoplasms and despite advances in surgery, radiotherapy, chemotherapy and targeted agents, patients still suffer from a poor prognosis. The incidence of these tumours has markedly increased over the past decades and in 2012 pancreatic cancers of all types were the seventh most common cause of cancer deaths, resulting in 330.000 deaths globally [1]. The overall 5-year survival is 5 %, all stages of the disease combined, and the median survival has been reported to be 5–8 months [2-4]. There are no early detection tests and most patients with localized disease have no recognizable symptoms or signs, resulting in late diagnosis in the majority of cases. Only15-20 % of the tumours are resectable at presentation [5], resectability often being limited by early local invasion of the surrounding anatomical structures, such as mesenteric arteries, or distant metastasis. There are two major morphological types of periampullary adenocarcinomas, i.e. pancreatobiliary (PB-type) adenocarcinomas (including pancreatic cancer, distal bile duct cancer, and some of the ampullary carcinomas) and intestinal type (I-type) periampullary adenocarcinomas (including duodenal carcinoma and some of the ampullary carcinomas). Morphological type seems to provide more important prognostic information in resected periampullary carcinoma than the tumour origin, with PB-type tumours being associated with significantly shorter survival rates than I-type tumours [6, 7]. The present diagnostic and prognostic information provided by histopathological parameters is far from sufficient, strongly implicating the need for additional molecular-based biomarkers to better define clinically relevant subgroups of these tumours for improved treatment stratification.

Podocalyxin-like protein (PODXL) is a member of the CD34 family of transmembrane sialomucins. PODXL is expressed on the apical surface of glomerular epithelial cells and podocytes [8], where it plays an integral role in maintaining adequate filtration [9], and it is also expressed on vascular endothelia [10] and hematopoietic stem cells [11, 12]. PODXL is upregulated in several types of cancer, and strong expression, in particular in the cell membrane, has been demonstrated to signify more aggressive tumours and poor prognosis in e.g. breast cancer [13], colorectal cancer [14-17] ovarian cancer [18], urinary bladder cancer [19], and glioblastoma [20].

PODXL has been found to be more frequently expressed (44 %) in pancreatic ductal adenocarcinoma as compared with other types of adenocarcinomas of the gastrointestinal and biliary tracts [21]. In another study, sialofucosylated PODXL was demonstrated to be a functional E- and L-selectin ligand expressed by metastatic pancreatic cancer cells *in vitro,* and was also found to be overexpressed, with membranous localization, in 69 % of 105 pancreatic ductal adenocarcinomas [22]. To our knowledge, the prognostic or predictive impact of PODXL expression in pancreatic or periampullary adenocarcinoma has not yet been described. The aim of the present study was therefore to examine the clinicopathological correlates, prognostic and predictive significance of tumour-specific PODXL expression in a retrospective cohort of pancreatic and periampullary adenocarcinoma, with particular reference to morphological subtypes thereof.

## Methods

### Patients

The study consists of a retrospective consecutive cohort of 175 patients with primary periampullary adenocarcinomas, surgically treated with pancreaticoduodenectomy at the University hospitals of Lund and Malmö, Sweden, from January 1 2001 until December 31 2011 [23-25]. Out of 175 cases in the entire cohort, there were 110 pancreatobiliary-type and 65 intestinal-type adenocarcinomas. Survival data were collected from the Swedish National Civil Register. Follow-up started at the date of surgery and ended at death, at 5 years after surgery or at December 31 2013, whichever came first. Information on neoadjuvant and adjuvant treatment and recurrence was obtained from patient records.

All haematoxylin & eosin stained slides from all cases were re-evaluated by one pathologist (JEL), blinded to the original report and outcome. The decision on tumour origin and morphological type was based on several criteria, as previously described [23].

The study has been approved by the Ethics Committee of Lund University (ref nr 445/07).

### Tissue microarray construction

Tissue microarrays (TMAs) were constructed using a semi-automated arraying device (TMArrayer, Pathology Devices, Westminister, MD, USA). A standard set of three tissue cores (1 mm) were obtained from each of the 175 primary tumours and from lymph node metastases from 105 of the cases, whereby one to three lymph node metastases were sampled in each case. Paired samples with non-malignant pancreatic tissue from the resection specimens were also obtained from 50 of the cases, using a standard set of two 1 mm tissue cores.

### Immunohistochemistry and staining evaluation

For immunohistochemical analysis of PODXL expression, 4 μm TMA-sections were automatically pre-treated using the PT Link system and then stained in an Autostainer Plus (DAKO; Glostrup, Copenhagen, Denmark) with the affinity-purified polyclonal, monospecific PODXL antibody (HPA002110; Atlas Antibodies AB, Stockholm, Sweden) diluted 1: 250. This antibody, originally generated within the Human Protein Atlas (HPA) project, has also been used in and validated in several previous biomarker studies on e.g. colorectal, bladder, pancreatic and testicular cancer [14, 19, 22, 26]. The expression of PODXL was recorded as negative (0), weak cytoplasmic positivity in any proportion of cells (1), moderate cytoplasmic positivity in any proportion of cells (2), distinct membranous positivity in < = 50 % of cells (3) and distinct membranous positivity in >50 % of cells (4) as previously described [14-16, 19]. Staining of PODXL was evaluated by two independent observers (MH and KJ) who were blinded to clinical and outcome data. Scoring differences were discussed in order to reach consensus.

### Statistical analysis

Chi square test was applied to analyse the relationship between PODXL expression and clinicopathological parameters. Two patients with PB-type adenocarcinomas who had received neoadjuvant chemotherapy were excluded from the correlation and survival analyses. Three additional patients were excluded from the survival analyses; two with I-type adenocarcinomas who died within one month from surgery due to complications and one with PB-type adenocarcinoma who emigrated 5 months after surgery.

Kaplan Meier analysis and log rank test were applied to estimate differences in 5-year overall survival (OS) and recurrence-free survival (RFS) in strata according to membranous and non-membranous PODXL expression. Hazard ratios (HR) for death and recurrence within 5 years were calculated by Cox regression proportional hazard´s modelling in unadjusted analysis and in a multivariable model adjusted for age, sex, T-stage, N-stage, differentiation grade, lymphatic invasion, vascular invasion, perineural invasion, infiltration in peripancreatic fat, resection margins, tumour origin, and adjuvant chemotherapy. A backward conditional method was used for variable selection in the adjusted model. To estimate the interaction effect between adjuvant treatment and PODXL expression in order to measure any possible difference in treatment effect based on PODXL expression, the following interaction variable was constructed; any adjuvant treatment (+/−) × PODXL (+/−).

All tests were two sided. *P-values* <0.05 were considered significant. All statistical analyses were performed using IBM SPSS Statistics version 20.0 (SPSS Inc., Chicago, IL, USA).

## Results

### PODXL expression in non-malignant pancreas, primary tumours and lymph node metastases

**Fig. 1:**
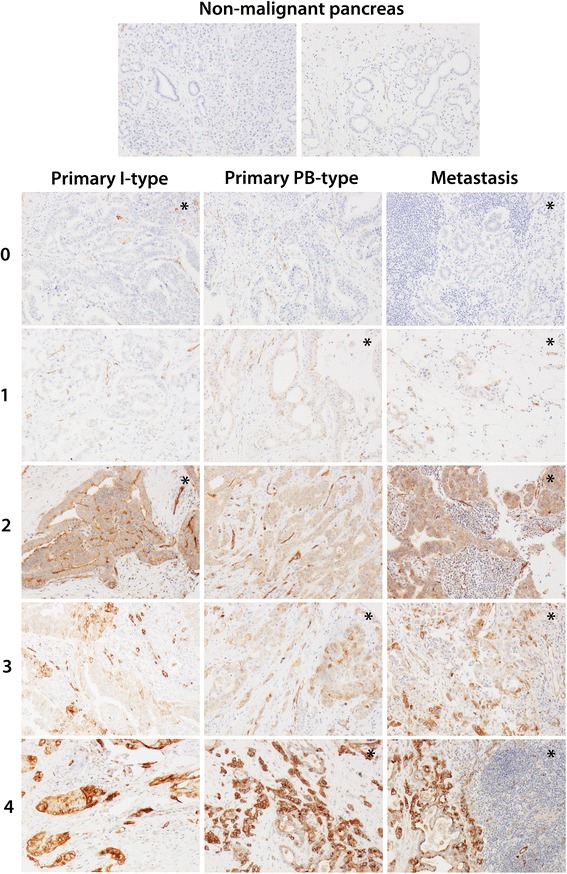
Sample immunohistochemical images. Immunohistochemical images of PODXL- negative non-malignant pancreatic tissue from two cases (*top row*), primary intestinal-type (*I-type*) tumours (*left column*), primary pancreatobiliary-type (*PB-type*) tumours (*mid-column*) and metastases (*right column*) with different PODXL staining scores (*0–4*). Asterisks indicate paired samples; i.e. from the same case/resection specimen. Score 0 = negative staining, score 1 = weak cytoplasmic positivity in any proportion of cells, score 2: moderate-strong cytoplasmic positivity in any proportion of cells, score 3: distinct membranous positivity in < = 50 % of cells and score 4 = distinct membranous positivity in >50 % of cells. All images with 10X original magnification

**Fig. 2:**
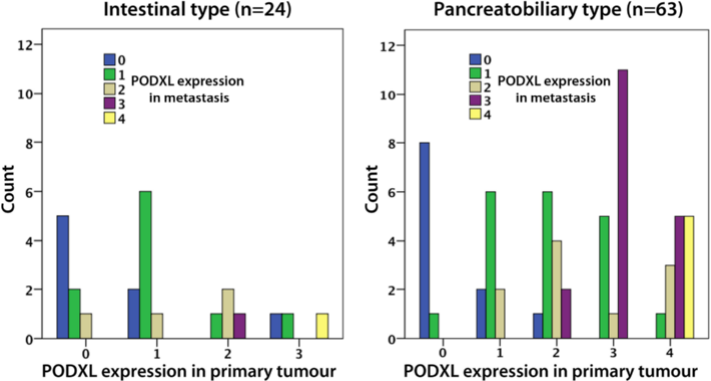
PODXL expression in primary tumours and metastases

Sample immunohistochemical images of PODXL expression are shown in Fig. 1. PODXL expression could be assessed in in 63/65 (96.9 %) primary I-type carcinomas and 24/30 (80.0 %) lymph node metastases, and in 107/108 (99.1 %) primary PB-type carcinomas and 63/75 (84.0 %) corresponding lymph node metastases. PODXL expression could be assessed in 49/50 (98 %) paired non-malignant samples, all displaying negative or very weak PODXL expression in acini and ducts. The distribution of PODXL expression in primary tumours and metastases, which did not differ significantly, by histological subtype, is shown in Fig. 2. Membranous PODXL expression was denoted in 11/63 (17.5 %) primary and 2/24 (8.3 %) metastatic I-type carcinomas, and in 53/107 (49.5 %) primary and 23/63 (36.5 %) metastatic PB-type carcinomas. In I-type tumours, membranous PODXL in the metastasis was seen in 1/21 (4.8 %) cases denoted as having non-membranous expression in the primary tumour, and non-membranous PODXL expression in the metastasis was denoted in 2/3 (66.7 %) cases with primary tumours displaying membranous PODXL expression. In PB-type tumours, the number of cases with non-membranous to membranous conversion was 2/32 (6.2 %) and with membranous to non-membranous conversion 10/31 (32.3 %). In all further statistical analyses, a dichotomized variable of non-membranous (score 0, 1, 2) versus membranous (score 3, 4) PODXL expression in the primary and/or metastatic component is applied. According to this combined variable, 12/63 (19.0 %) I-type cases and 55/107 (51.4 %) PB-type cases displayed membranous PODXL expression in any component.

### Associations of PODXL expression with clinicopathological factors

The associations between PODXL expression and clinicopathological factors in I-type and PB-type tumours, respectively, are shown in Table 1. In I-type tumours, there were no significant associations between PODXL expression and clinicopathological factors. In PB-type tumours, membranous PODXL expression was significantly associated with female sex (*p* = 0.005), with location to the pancreas (*p* = 0.005), and with poor differentiation grade (*p* = 0.044). There was no statistically significant association between PODXL expression and other clinicopathological factors including age at diagnosis, tumour size, T-stage, N-stage, resection margins, presence of vascular- lymphatic and neural invasion and growth in peripancreatic fat.

**Table 1:** Associations between membranous and non-membranous PODXL expression with clinciopathological parameters in intestinal type and pancreatobiliary type tumours, respectively

	Intestinal type			Pancreatobiliary type		
	PODXL NM	PODXL M	P	PODXL NM	PODXL M	P
	(*n* = 51)	(*n* = 12)		(*n* = 52)	(*n* = 55)	
Age						
(median, range)	66.0 (38.0–83.0)	67.5 (44.0–74.0)	0.972	66.0 (44.0–81.0)	68.0 (44.0–81.0)	0.613
Sex						
Women	28 (82.4)	6 (17.6)	0.761	17 (34.0)	33 (66.0)	0.005
Men	23 (79.3)	6 (20.7)		35 (61.4)	22 (38.6)	
Tumour origin						
Duodenum	12 (85.7)	2 (14.3)	0.610			
Ampulla intestinal type	39 (79.6)	10 (20.4)				
Ampulla pancreatobiliary type				14 (73.7)	5 (26.3)	0.005
Distal bile duct				23 (51.1)	22 (48.9)	
Pancreas				15 (34.9)	28 (65.1)	
Tumour size mm						
(median, range)	30.0 (5.0–90.0)	26.5 (12.0–40.0)	0.923	30.0 (5.0–70.0)	30.0 (15.0–70.0)	0.313
Differentiation grade						
Well-moderate	26 (83.9)	5 (16.1)	0.565	24 (61.5)	15 (38.5)	0.044
Poor	25 (78.1)	7 (21.9)		28 (41.2)	40 (58.8)	
T-stage						
T1	4 (100.0)	0 (0.0)	0.246	2 (100.0)	0 (0.0)	0.392
T2	9 (81.8)	2 (18.2)		4 (40.0)	6 (60.0)	
T3	21 (84.0)	4 (16.0)		34 (43.6)	44 (56.4)	
T4	17 (73.9)	6 (26.1)		12 (70.6)	5 (29.4)	
N-stage						
N0	28 (84.8)	5 (5.2)	0.936	17 (56.7)	13 (43.3)	0.315
N1	13 (68.4)	6 (31.6)		21 (46.7)	24 (53.3)	
N2	10 (90.9)	1 (9.1)		14 (43.8)	18 (56.2)	
Margins						
R0	15 (88.2)	2 (11.8)	0.375	3 (50.0)	3 (50.0)	0.944
R1-Rx	36 (78.3)	10 (21.7)		49 (48.5)	52 (51.5)	
Perineural growth						
No	38 (86.4)	6 (13.6)	0.099	14 (63.6)	8 (36.4)	0.115
Yes	13 (68.4)	6 (31.6)		38 (44.7)	47 (55.3)	
Invasion of lymphatic vessels						
No	26 (89.7)	3 (10.3)	0.107	16 (50.0)	16 (50.0)	0.850
Yes	25 (73.5)	9 (26.5)		36 (48.0)	39 (52.0)	
Invasion of blood vessels						
No	48 (82.8)	10 (17.2)	0.217	35 (50.0)	35 (50.0)	0.691
Yes	3 (60.0)	2 (40.0)		17 (45.9)	20 (54.1)	
Growth in peripancreatic fat						
No	35 (85.4)	6 (14.6)	0.227	13 (59.1)	9 (40.9)	0.271
Yes	16 (72.7)	6 (27.3)		39 (45.9)	46 (54.1)	
Adjuvant chemotherapy						
None	40 (88.9)	5 (11.1)	0.145	23 (46.0)	27 (54.0)	0.348
5-FU-analogue	2 (40.0)	3 (60.0)		5 (62.5)	3 (37.5)	
Gemcitabine	4 (57.1)	3 (42.9)		20 (45.5)	24 (54.5)	
Gemcitabine + capecitabine	1 (100.0)	0 (0.0)		1 (50.0)	1 (50.0)	
Oxaliplatin + 5-FU analogue	4 (100.0)	0 (0.0)		1 (100.0)	0 (0.0)	
Gemcitabine + oxaliplatin	0 (0.0)	1 (100.0)		2 (100.0)	0 (0.0)	

*M* membranous PODXL expression, *NM* non-membranous PODXL expression

*R0* radical resection, *R1* non-radical resection, *RX* uncertain resection margins

### Prognostic and potential predictive value of PODXL expression

**Fig. 3:**
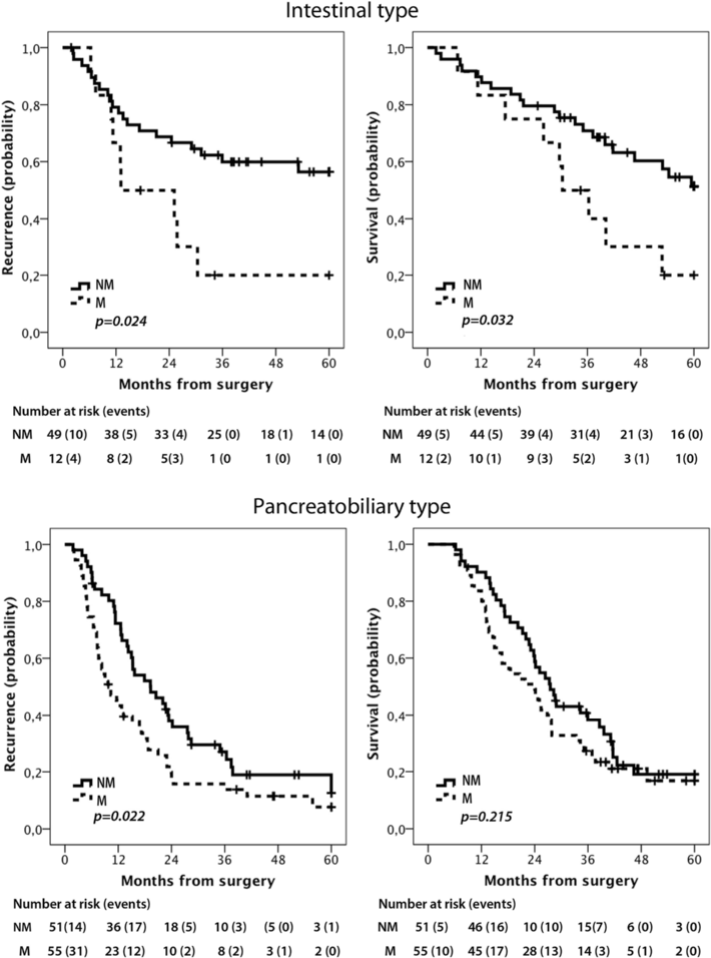
Survival according to membranous versus non-membranous PODXL expression. Kaplan-Meier estimates of recurrence free and 5-year overall survival in patients with intestinal and pancreatobiliary type tumours, respectively. NM= non-membranous, M= membranous PODXL expression

As demonstrated in Fig. 3, Kaplan-Meier analysis revealed significant associations of membranous PODXL expression with a reduced RFS (logrank *p* = 0.024) and OS (logrank *p* = 0.032) in I-type tumours and with a reduced RFS (logrank *p* = 0.022) but not OS in PB-type tumours. These associations were confirmed in univariable Cox regression analysis for both RFS (Table 2) and OS (Table 3) in I-type tumours (HR = 2.44, 95 % CI 1.10–5.44, and HR = 2.32, 95 % CI 1.05–5.12, respectively) and for RFS (Table 2), but not OS (Table 3) in PB-type tumours (HR = 1.63, 95 % CI 1.07–2.49, logrank *p* = 0.022). In multivariable analysis, PODXL remained a significant prognostic factor only in I-type tumours (HR = 5.12, 95 % CI 1.43–18.31 for RFS, Table 2, and HR = 7.31, 95 % CI 2.12–25.16 for OS, Table 3).

**Table 2:** Unadjusted and adjusted hazard ratios for recurrence within five years in intestinal and pancreatobiliary type tumours

	Intestinal type			Pancreatobiliary type		
		Unadjusted	Adjusted		Unadjusted	Adjusted
	n(events)	HR(95%CI)	HR(95%CI)	n(events)	HR(95%CI)	HR(95%CI)
Age						
Continuous	61 (29)	1.00 (0.96–1.03)	1.07 (1.02–1.13)	106 (88)	0.98 (0.96–1.01)	1.00 (0.96–1.03)
Gender						
Female	34 (11)	1.00	1.00	50 (42)	1.00	1.00
Male	27 (18)	2.31 (1.08–4.94)	2.40 (0.94–6.14)	56 (46)	1.06 (0.70–1.61)	0.88 (0.53–1.49)
Tumour origin						
Duodenum	13 (4)	1.00	1.00		_	_
Ampulla-Intestinal type	48 (25)	2.18 (0.76–6.27)	6.82 (1.32–35.12)		_	_
Ampulla-Pancreatobiliary type		_	_	19 (16)	1.00	1.00
Distal Bile duct		_	_	44 (38)	1.10 (0.61–1.98)	1.52 (0.78–2.94)
Pancreas		_	_	43 (34)	1.06 (0.58–1.93)	0.93 (0.49–1.77)
Tumour size						
Continuous	61 (30)	1.00 (0.98–1.02)	1.04 (1.00–1.09)	106 (88)	1.03 (1.02–1.05)	1.02 (0.99–1.04)
T-stage						
T1	4 (1)	1.00	1.00	2 (1)	1.00	1.00
T2	10(3)	1.28 (0.13–12.30)	3.71 (0.34–40.64)	10 (6)	1.61 (0.19–13.36)	0.66 (0.07–6.08)
T3	25 (9)	1.86 (0.24–14.71)	5.72 (0.50–65.06)	77 (66)	4.67 (0.64–33.96)	1.21 (0.15–9.92)
T4	22 (16)	5.44 (0.72–41.21)	6.36 (0.21–195.20)	17 (15)	4.31 (0.56–33.10)	1.83 (0.08–40.27)
N-stage						
N0	33 (11)	1.00	1.00	29 (21)	1.00	1.00
N1 (metastasis in 1–3 lgl)	19 (11)	2.07 (0.90–4.78)	1.00 (0.37–2.70)	45 (37)	2.17 (1.25–3.78)	2.04 (1.13–3.67)
N2 (metastasis in 4 or more lgl)	9 (7)	4.06 (1.55–10.59)	6.88 (1.81–26.15)	32 (30)	3.11 (1.72–5.61)	2.61 (1.42–4.83)
Differentiation grade						
Well-moderate	30 (11)	1.00	1.00	39 (29)	1.00	1.00
Poor	31 (18)	2.16 (1.02–4.57)	1.38 (0.40–4.79)	67 (59)	2.32 (1.45–3.71)	2.02 (1.20–3.39)
Involved margins, status						
R0	17 (3)	1.00	1.00	6 (4)	1.00	1.00
R1 & Rx	44 (26)	4.51 (1.36–14.94)	2.23 (0.62–8.03)	100 (84)	2.31 (0.84–6.36)	2.39 (0.84–6.76)
Lymphatic growth						
Absent	28 (5)	1.00	1.00	32 (23)	1.00	1.00
Present	33 (24)	6.16 (2.34–16.19)	6.19 (1.76–21.82)	74 (65)	1.77 (1.09–2.88)	1.05 (0.59–1.85)
Vascular growth						
Absent	56 (24)	1.00	1.00	70 (55)	1.00	1.00
Present	5 (5)	8.16 (2.86–23.30)	1.62 (0.39–6.65)	36 (33)	2.30 (1.47–3.61)	2.08 (1.28–3.36)
Perineural growth						
Absent	42 (15)	1.00	1.00	22 (14)	1.00	1.00
Present	19 (14)	2.72 (1.31–5.66)	1.01 (0.27–3.81)	84 (74)	2.93 (1.57–5.46)	2.04 (1.06–3.90)
Growth in peripancreatic fat						
Absent	40 (12)	1.00	1.00	22 (13)	1.00	1.00
Present	21 (17)	4.74 (2.23–10.10)	3.60 (1.43–9.07)	84 (75)	2.60 (1.42–4.75)	1.45 (0.76–2.77)
Adjuvant treatment						
No	43 (21)	1.00	1.00	49 (40)	1.00	1.00
Yes	18 (8)	0.87 (0.38–1.96)	0.12 (0.04–0.44)	57 (48)	1.08 (0.70–1.65)	0.89 (0.54–1.49)
PODXL expression						
Non-membranous	49 (20)	1.00	1.00	51 (40)	1.00	1.00
Membranous	12 (9)	2.44 (1.10–5.44)	5.12 (1.43–18.31)	55 (88)	1.63 (1.07–2.49)	1.53 (0.99–2.38)

*R0* radical resection, *R1* non-radical resection, *RX* uncertain resection margins

**Table 3:** Unadjusted and adjusted hazard ratios for death within five years in intestinal and pancreatobiliary type tumours

	Intestinal type			Pancreatobiliary type		
		Unadjusted	Adjusted		Unadjusted	Adjusted
	n(events)	HR(95%CI)	HR(95%CI)	n(events)	HR(95%CI)	HR(95%CI)
Age						
Continuous	61 (30)	1.02 (0.98–1.06)	1.07 (1.02–1.13)	106 (82)	0.99 (0.96–1.02)	1.01 (0.98–1.05)
Gender						
Female	34 (13)	1.00	1.00	50 (36)	1.00	1.00
Male	27 (17)	1.85 (0.89–3.84)	2.12 (0.86–5.22)	56 (46)	1.20 (0.78–1.87)	1.22 (0.76–1.95)
Tumour origin						
Duodenum	13 (5)	1.00	1.00		_	_
Ampulla-Intestinal type	48 (25)	1.49 (0.57–3.88)	7.77 (1.86–32.39)		_	_
Ampulla-Pancreatobiliary type		_	_	19 (16)	1.00	1.00
Distal Bile duct		_	_	44 (32)	0.74 (0.40–1.34)	1.03 (0.50–2.15)
Pancreas		_	_	43 (34)	0.91 (0.50–1.65)	1.06 (0.52–2.19)
Tumour size						
Continuous	61 (30)	1.00 (0.98–1.03)	1.05 (1.01–1.10)	106 (82)	1.03 (1.01–1.05)	1.01 (0.99–1.04)
T-stage						
T1	4 (2)	1.00	1.00	2 (1)	1.00	1.00
T2	10(3)	0.65 (0.11–3.88)	0.55 (0.07–4.50)	10 (6)	1.43 (0.17–11.85)	0.77 (0.08–7.56)
T3	25 (9)	0.94 (0.20–4.37)	1.49 (0.19–11.34)	77 (60)	2.95 (0.41–21.34)	0.84 (0.10–7.08)
T4	22 (16)	2.55 (0.58–11.15)	1.88 (0.19–18.25)	17 (15)	3.77 (0.50–28.71)	2.12 (0.09–48.78)
N-stage						
N0	33 (15)	1.00	1.00	29 (18)	1.00	1.00
N1 (metastasis in 1–3 lgl)	19 (9)	1.17 (0.51–2.68)	0.55 (0.20–1.50)	45 (37)	2.41 (1.35–4.29)	2.85 (1.57–5.17)
N2 (metastasis in 4 or more lgl)	9 (6)	2.08 (0.80–5.37)	8.96 (2.47–32.51)	32 (27)	2.59 (1.40–4.78)	2.45 (1.30–4.63)
Differentiation grade						
Well-moderate	30 (12)	1.00	1.00	39 (24)	1.00	1.00
Poor	31 (18)	1.98 (0.95–4.11)	2.16 (0.77–6.03)	67 (58)	2.44 (1.50–3.95)	2.13 (1.28–3.54)
Involved margins, status						
R0	17 (4)	1.00	1.00	6 (2)	1.00	1.00
R1 & Rx	44 (26)	2.56 (0.89–7.36)	0.46 (0.12–1.69)	100 (80)	3.49 (0.86–14.25)	2.57 (0.62–10.60)
Lymphatic growth						
Absent	28 (7)	1.00	1.00	32 (22)	1.00	1.00
Present	33 (23)	3.61 (1.55–8.44)	5.85 (1.93–17.77)	74 (60)	1.51 (0.92–2.48)	0.96 (0.55–1.70)
Vascular growth						
Absent	56 (25)	1.00	1.00	70 (47)	1.00	1.00
Present	5 (5)	7.78 (2.74–22.11)	1.70 (0.40–7.31)	36 (35)	2.39 (1.54–3.72)	2.45 (1.54–3.87)
Perineural growth						
Absent	42 (17)	1.00	1.00	22 (14)	1.00	1.00
Present	19 (13)	2.15 (1.04–4.44)	3.81 (1.55–9.37)	84 (68)	1.88 (1.05–3.38)	0.92 (0.48–1.76)
Growth in peripancreatic fat						
Absent	40 (14)	1.00	1.00	22 (14)	1.00	1.00
Present	21 (16)	3.49 (1.68–7.25)	0.75 (0.06–9.94)	84 (68)	1.80 (1.00–3.25)	1.25 (0.64–2.43)
Adjuvant treatment						
No	43 (24)	1.00	1.00	49 (39)	1.00	1.00
Yes	18 (6)	0.60 (0.25–1.47)	0.03 (0.01–0.16)	57 (43)	0.90 (0.58–1.39)	0.67 (0.43–1.04)
PODXL expression						
Non-membranous	49 (21)	1.00	1.00	51 (38)	1.00	1.00
Membranous	12 (9)	2.32 (1.05–5.12)	7.31 (2.12–25.16)	55 (44)	1.32 (0.85–2.03)	1.10 (0.67–1.81)

*R0* radical resection, *R1* non-radical resection, *RX* uncertain resection margins

**Fig. 4:**
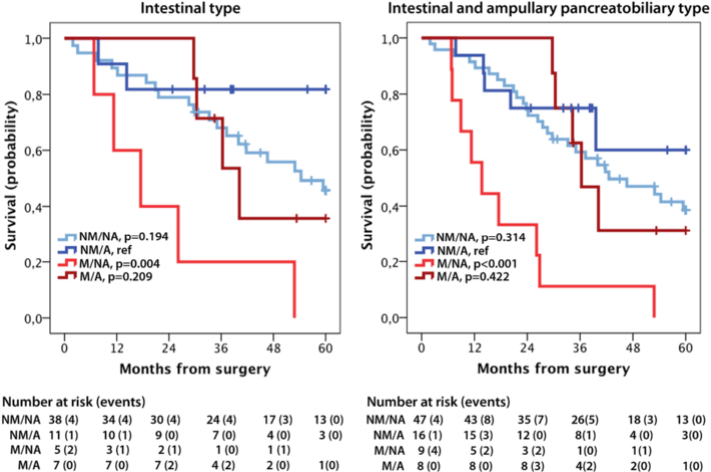
Survival according to PODXL expression and adjuvant chemotherapy. Kaplan-Meier estimates of 5-year overall survival in combined strata of membranous (*M*)/ non-membranous (*NM*) PODXL expression and adjuvant (*A*) /no adjuvant (*NA*) chemotherapy in patients with intestinal type tumours and intestinal type + ampullary pancreatobiliary type tumours, respectively. NM= non-membranous, M= membranous PODXL expression

Next, we examined the potential predictive impact of PODXL expression on survival in strata according to adjuvant treatment. As demonstrated in Fig. 4, patients with I-type tumours displaying membranous PODXL expression had a significant beneficial effect of adjuvant chemotherapy regarding 5-year OS. When ampullary PB-type tumours, expressing membranous PODXL in a similar proportion to I-type tumours, were included in the analysis, the beneficial value of adjuvant chemotherapy was even more pronounced (Fig. 4). Hazard ratios for 5-year OS according to adjuvant treatment and PODXL expression are shown in Additional file 1. The results demonstrate that survival did not differ significantly by membranous PODXL-expression in patients with I-type tumours or the extended group of I-type + ampullary PB-type tumours having received adjuvant chemotherapy. In contrast, the adverse prognostic impact of membranous PODXL expression was even more evident in patients not receiving adjuvant chemotherapy compared to the entire group (unadjusted HR = 4.38, 95 % CI 1.57–12.18 in I-type tumours, and unadjusted HR = 7.13; 95 % CI 2.64–19.26 in I-type + ampullary PB-type tumours). These associations remained significant in multivariable analysis, but there was no significant treatment interaction (Additional file 1). These associations were not significant in relation to RFS (data not shown) or in PB-type tumours (data not shown). The prognostic and predictive impact of membranous PODXL expression was similar when only its expression in the primary tumour was considered (data not shown). The prognostic value of the full range of PODXL scores (0–4) in relation to RFS and OS, in the entire cohort and by morphological subtype, is shown in Additional file 2. All survival analyses were also performed using a dichotomized variable of score 0–1 vs 2–4, with allover less significant results (data not shown).

## Discussion

Pancreatic cancer is an extremely lethal type of cancer. On average, patients die from the disease within 6 months from diagnosis. Therefore it is of uttermost importance to find both predictive and prognostic factors so as to improve treatment. The results from this study provide a first demonstration of the prognostic and potential predictive value of PODXL in pancreatic, distal bile duct, ampullary and duodenal adenocarcinoma. PODXL-expression was found to be significantly higher in PB-type as compared with I-type tumours, with the exception for ampullary PB-type tumours. These findings are in line with the expected and provide further evidence of PODXL being associated with a more aggressive tumour phenotype and a biomarker of poor prognosis in human cancer.

The study cohort encompasses a retrospective cohort of 110 pancreatobiliary-type and 65 intestinal-type adenocarcinomas, including paired normal tissue and lymph node metastases from a subset of cases, thus providing a thorough characterization of PODXL expression in a wide range of periampullary adenocarcinomas. In the present study, membranous PODXL expression was denoted in 49.5 % of primary PB-type carcinomas, which is somewhat lower than in the previous study by Dallas et al., including tumours from 105 cases assembled in TMAs, wherein membranous PODXL expression was found in 69 % of the cases [22]. In primary I-type carcinomas, membranous PODXL expression was denoted in 17.5 %, which is well in line with previous TMA-based studies on colorectal cancer wherein membranous expression was found in 13.4 % and 9.6 % respectively [14, 15]. This observation further supports the theory that I-type carcinomas of the pancreatic region resemble tumours with colorectal origin in a stronger way than PB-types. In line with the study by Ney et al., PODXL was negative or only weakly expressed in normal pancreatic parenchyma from the resection specimens [21].

In a previous study on colorectal cancer, wherein a PODXL expression was compared in full-face sections from 31 primary tumours and all available lymph node metastases (*n* = 140), there was an excellent concordance in that all primary tumours with non-membranous PODXL expression had metastases with non-membranous expression, whereas a few primary tumours with membranous PODXL expression had a varying proportion of metastatic lymph nodes with membranous and non-membranous PODXL expression [16]. These findings led to the conclusion that for prognostic or predictive purposes, analysis of the primary tumour would be sufficient [16]. In the present study, although negative conversion of membranous PODXL expression from primary tumour to lymph node metastasis was far more common than positive conversion, a few cases displayed the latter phenomenon. Of note, all analyses were based on TMA-samples, and therefore, future studies based on full-face sections are warranted to further examine the rate of positive conversion of membranous PODXL expression in pancreatic and periampullary cancers, so as to determine whether biomarker analysis of the primary tumour will be sufficient in the clinical setting.

In the present study membranous PODXL expression was an independent predictor of reduced 5-year overall and recurrence-free survival in I-type but not in PB-type tumours, although there was a significant association between membranous PODXL expression and a reduced RFS in the latter in unadjusted analysis. These findings are well in line with previous publications regarding the prognostic significance of PODXL expression in several other major types of cancer [13-15, 18-20]. In addition, and importantly, we found that patients with I-type tumours displaying membranous PODXL had a beneficial effect of adjuvant chemotherapy. When ampullary PB-type tumours, expressing membranous PODXL in a similar proportion to I-type tumours, were included in the analysis, the effect by adjuvant chemotherapy was even more pronounced. This supports earlier data that patients with PODXL positive tumours benefit from adjuvant chemotherapy, irrespective of treatment regime, as seen in colorectal cancer [14, 15]. Moreover, these findings indicate that I-type tumours with high expression of PODXL are more likely to benefit from adjuvant therapy than PB-type tumours. Today, all patients with pancreatic and periampullary adenocarcinoma are recommended adjuvant treatment. Since adjuvant treatment often is associated with toxicity and adverse side effects, it is important to identify novel predictive and prognostic factors, such as PODXL, to support and improve clinical decisions. Therefore, the results from the present study indicate that PODXL could be used as a predictive marker for adjuvant treatment of periampullary cancer with intestinal morphology, and possibly also ampullary PB-type tumours. Of note, given the retrospective nature of the present study, the term “predictive” should be applied with caution. It must however be pointed out that the study cohort encompasses a consecutive series of clinically and histopathologically well-annotated pancreatoduodenectomy cases, of which only approximately half have been given adjuvant chemotherapy, which should allow for a fairly good assessment of both prognostic and predictive biomarkers even in the retrospective setting. Thus, the herein observed prognostic and potential predictive value of PODXL, in particular in I-type tumours, is of potential clinical relevance and merits further study in additional retrospective cohorts as well as in a controlled, prospective trial. Targeting PODXL with monoclonal antibodies may also be a future treatment option [27].

Membranous PODXL expression was considerably higher in PB-type as compared with I-type tumours, which is in line with the former being clinically more aggressive. In PB-type tumours, the prognostic value of PODXL was only significant for RFS, and not after adjustment for other clinicopathological factors, and there was no evident predictive value. The choice of prognostic cutoff, i.e. membranous vs non-membranous PODXL expression, can be considered appropriate for the herein used antibody, since the same antibody and cutoff has been used in the previous study on pancreatic cancer by Dallas et al.[22] and since this dichotomization yielded the strongest prognostic and predictive value. It is however noteworthy that the category of tumours with moderate-strong cytoplasmic staining (score 2) is a somewhat ambiguous group with an intermediate prognosis, undoubtedly harbouring some cases with a prognosis equally poor to cases with membranous PODXL expression. While it is possible that in some of these cases, the presence of membranous expression may be masked by a strong cytoplasmic expression, this category of tumours may also constitute a different biological entity, possibly constituting an “intermediate” between tumours with negative/weak and membranous PODXL expression. In a comparative study on colorectal cancer, membranous expression of the herein used antibody and cytoplasmic expression of an in-house generated antibody were both found to be independent predictors of poor prognosis, and combined use of the antibodies was found to detect a group with an even worse prognosis [28].

Previous studies have demonstrated PODXL to be a functional ligand of E- and L- selectins in pancreatic cancer suggesting that its expression may promote haemotogenic spread of metastases by facilitating binding of circulating tumour cells to selectin-expressing host cells [22]. These findings further support the theory of PODXL overexpression being associated with more aggressive tumours [22]. Moreover, similar to the situation in colorectal [14, 15, 16] and urinary bladder [19] cancer, PODXL expression was observed predominantly on the invasive tumour front, also suggesting its importance in the metastatic spread of the disease. Of note, in the study on bladder cancer, the herein used polyclonal antibody was compared with two other monoclonal antibodies, all showing 100 % concordance regarding the detection of membranous PODXL expression, whereas the degree of cytoplasmic expression detected by the monoclonal antibodies was substantially weaker [19].

Our results are derived from TMA-based analyses on retrospectively collected tumour samples. Of note, the TMA-technique was also used in the study by Dallas et al. [22]. For characterization of key molecular alterations and expression of investigative biomarkers in tumours from large patient cohorts, whether retrospectively or prospectively defined, the TMA-technology is essential [29]. However, some limitations related to the TMA-technique must be considered, most importantly its ability to accurately reflect the expression of heterogeneously expressed markers. To compensate for this one needs to ensure that tumour cores are sampled from different regions of the tumour. In the present study, the cores from the primary tumour were, whenever possible, obtained from different donor blocks, and different lymph nodes were sampled in cases with more than one metastatic node.

## Conclusions

Membranous expression of PODXL is significantly higher in pancreatobiliary type as compared with intestinal type periampullary adenocarcinomas and an independent factor of poor prognosis in the latter. The herein presented results also indicate a beneficial effect of adjuvant chemotherapy on intestinal type tumours with membranous PODXL expression, suggesting the potential utility of PODXL as a biomarker for improved treatment stratification of these patients.

## Additional files

Additional file 1:
**Cox proportional hazards analysis of the impact of PODXL expression on overall survival according to adjuvant treatment intestinal-type and intestinal-type + ampullary pancreatobiliary-type adenocarcinomas.**
Additional file 2:
**Survival according to PODXL score. Kaplan-Meier estimates of recurrence free survival and 5-year overall survival, respectively, in (A,B) the entire cohort, (C, D) patients with intestinal type tumours and (E, F) patients with pancreatobiliary type tumours.** Score 0 = negative staining, score 1 = weak cytoplasmic positivity in any proportion of cells, score 2: moderate-strong cytoplasmic positivity in any proportion of cells, score 3: distinct membranous positivity in < = 50 % of cells and score 4 = distinct membranous positivity in >50 % of cells.

## Abbreviations

PODXL: Podocalyxin-like protein 1
PB-type: Pancreatobiliary type adenocarcinoma
I-type: Intestinal type adenocarcinoma
TMA: Tissue microarray
OS: Overall survival
RFS: Recurrence free survival
HR: Hazard ratio
CI: Confidence interval
