# Supplementary material for: Prognostic and predictive significance of podocalyxin-like protein expression in pancreatic and periampullary adenocarcinoma
Source: BMC Clin Pathol. 2015 May 30;15:10. doi: 10.1186/s12907-015-0009-1 (PMC4449563; doi:10.1186/s12907-015-0009-1)
Supplement: Additional file 1: — Cox proportional hazards analysis of the impact of PODXL expression on overall survival according to adjuvant treatment intestinal-type and intestinal-type + ampullary pancreatobiliary-type adenocarcinomas. [file 12907_2015_9_MOESM1_ESM.docx]

**Additional file 1. Cox proportional hazards analysis of the impact of PODXL expression on overall survival according to adjuvant treatment intestinal-type and intestinal-type + ampullary pancreatobiliary-type adenocarcinomas.**

| **Intestinal-type tumours** | **HR (95% CI)** | **Number (events)** |  |
| --- | --- | --- | --- |
| ***All*** | *unadjusted* |  |  |
| PODXL non-membranous | 1.00 | 49 (21) |  |
| PODXL membranous | 2.32 (1.05-5.12) | 12 (9) |  |
|  | adjusted |  |  |
| PODXL non-membranous | 1.00 | 49 (21) |  |
| PODXL membranous | 7.31 (2.12-25.16) | 12 (9) | P for interaction |
| ***No adjuvant treatment*** | *unadjusted* |  | *0.721* |
| PODXL non-membranous | 1.00 | 38 (19) |  |
| PODXL membranous | 4.38 (1.57-12.18) | 5 (5) |  |
|  | *adjusted* |  |  |
| PODXL non-membranous | 1.00 | 38 (19) |  |
| PODXL membranous | 64.31 (8.79-470.66) | 5 (5) |  |
| ***Any adjuvant treatment*** |  |  |  |
| PODXL non-membranous | 1.00 | 11 (2) |  |
| PODXL membranous | 2.84 (0.52-15.54) | 7 (4) |  |
| **Intestinal-type + ampullary pancreatobiliary-type tumours** |  |  |  |
| ***All*** | *unadjusted* |  |  |
| PODXL non-membranous | 1.00 | 63 (32) |  |
| PODXL membranous | 2.28 (1.21-4.31) | 17 (14) |  |
|  | *adjusted* |  |  |
| PODXL non-membranous | 1.00 | 63 (32) |  |
| PODXL membranous | 2.53 (1.20-5.31) | 17 (14) |  |
| ***No adjuvant treatment*** | *unadjusted* |  | *0.186* |
| PODXL non-membranous | 1.00 | 47 (27) |  |
| PODXL membranous | 4.47 (2.03-9.85) | 9 (9) |  |
|  | *adjusted* |  |  |
| PODXL non-membranous | 1.00 | 47 (27) |  |
| PODXL membranous | 7.13 (2.64-19.26) | 9 (9) |  |
| ***Any adjuvant treatment*** | *unadjusted* |  |  |
| PODXL non-membranous | 1.00 | 16 (5) |  |
| PODXL membranous | 1.65 (0.48-5.73) | 8 (5) |  |

Due to the small subgroups, multivariable analysis was not possible to perform in the categories with cases

receiving adjuvant treatment
