# Supplementary figures and images for: Prognostic and predictive significance of podocalyxin-like protein expression in pancreatic and periampullary adenocarcinoma
Source: BMC Clin Pathol. 2015 May 30;15:10. doi: 10.1186/s12907-015-0009-1 (PMC4449563; doi:10.1186/s12907-015-0009-1)

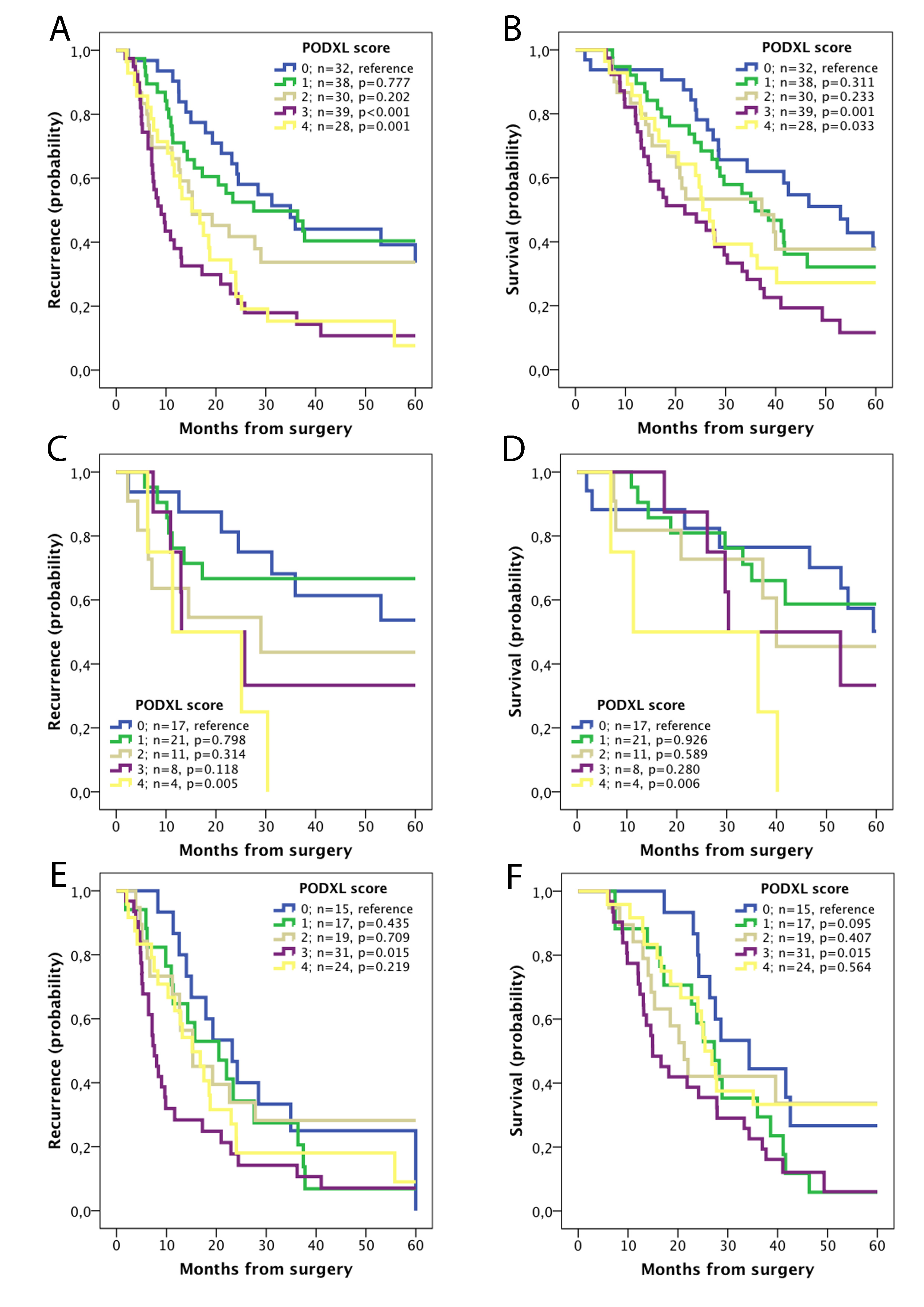

Supplement: Additional file 2: — Survival according to PODXL score. Kaplan-Meier estimates of recurrence free survival and 5-year overall survival, respectively, in (A,B) the entire cohort, (C, D) patients with intestinal type tumours and (E, F) patients with pancreatobiliary type tumours. Score 0 = negative staining, score 1 = weak cytoplasmic positivity in any proportion of cells, score 2: moderate-strong cytoplasmic positivity in any proportion of cells, score 3: distinct membranous positivity in < = 50 % of cells and score 4 = distinct membranous positivity in >50 % of cells. [file 12907_2015_9_MOESM2_ESM.jpeg]
